# Ability of donkey sperm to tolerate cooling: Effect of extender base and removal of seminal plasma on sperm parameters and fertility rates in mares

**DOI:** 10.3389/fvets.2022.1011899

**Published:** 2022-09-26

**Authors:** Mariana L. M. Gobato, Lorenzo G. T. M. Segabinazzi, Verônica F. C. Scheeren, Rafael S. Bandeira, Camila P. Freitas-Dell'Aqua, José A. Dell'Aqua, Frederico O. Papa

**Affiliations:** ^1^School of Veterinary Medicine and Animal Science, São Paulo State University (UNESP), Botucatu, São Paulo, Brazil; ^2^Ross University School of Veterinary Medicine, Basseterre, Saint Kitts and Nevis

**Keywords:** jack, stallion, artificial insemination, mule, equine

## Abstract

Artificial insemination using cooled-transported semen has marked importance in equine breeding programs around the world, and the high value of mules has generated avid interest in donkey semen biotechnology. However, donkey semen cools poorly in commercially available equine extenders. Therefore, this study aimed to develop approaches to improve the ability of donkey semen to tolerate cooling. Ejaculates of seven donkeys (*n* = 21) were cooled at 5°C for 48 h in three different extenders (milk-based, SM; sodium caseinate-based, SC; or egg yolk-based, EY) in the presence or absence of seminal plasma (centrifugation, C). Sperm motility, plasma membrane integrity (PMI), plasma membrane stability (PMS), mitochondrial membrane potential (HMMP), intracellular hydrogen peroxide (H_2_O_2_), and intracellular superoxide (${\text{O}}_{2}^{-}$) were assessed before, 24 h, and 48 h post-cooling. In addition, 15 mares (163 estrous cycles) were randomly inseminated with semen from two jacks (Jack 1, *n* = 90; Jack 2, *n* = 73) previously cooled for 24 h under one of the treatments (SM, SC, EY, SM-C, SC-C, or EY-C). Groups EY, SC-C, and EY-C (*P* < 0.05) demonstrated superior sperm analytical parameters to SM at 24 and 48 h. Centrifugation positively affected sperm analytical parameters in cooled donkey semen extended in SM and SC (*P* < 0.05). Mares bred with semen extended in SC (67%, 18/27), SC-C (89%, 24/27), EY (89%, 25/28), or EY-C (74%, 20/27) had significantly greater conception rates than mares bred with SM (33%, 9/27; *P* < 0.05). Mares bred with SM-C had intermediate conception rates (59%, 16/27). In conclusion, SC and EY improved the cooling ability and fertility of donkey semen in horse mares, and centrifugation positively affected donkey semen extended in SM.

## Introduction

Artificial insemination (AI) using cooled-transported semen has marked importance in equine breeding programs around the world (1). Although this technology is not yet employed large-scale across donkey species worldwide (1–3), the high value of mules has generated avid interest in donkey semen biotechnology within Brazil and the United States. Because of the lack of information regarding cooled donkey semen (4–8), guidelines intended for horse semen have been extrapolated to donkey species. However, this lack of species-specific processing of donkey semen may be responsible for the poor results seen in the donkey industry (2, 9, 10).

Different semen extender bases (e.g., skimmed milk, egg-yolk, defined milk proteins) are commercially available for cooling stallion semen. However, donkey semen does not clinically appear to tolerate cooling in these same commercially available milk- or milk-protein-based extenders (2). Additionally, even though few studies have compared semen extenders for cooling donkey sperm, there is presently no gold standard protocol for this species (4–8, 11–13). Clinical experience and previous studies suggest that donkey sperm needs an additional source of cholesterol to preserve sperm characteristics after cooling (2, 14). Some reports suggest that the poor cooling ability of jack semen may be circumvented by re-extending the semen in milk- or milk-protein-based extenders after removal of seminal plasma *via* centrifugation, or by adding 2% egg yolk to the extenders used (1, 5). However, both are time-consuming approaches, and producers processing donkey semen may not have a centrifuge or fresh eggs readily available.

A recent publication demonstrated that the addition of cholesterol-loaded cyclodextrin (CLC) to a skimmed milk-based (SM) extender may feasibly circumvent the poor ability of donkey semen to tolerate cooling and improve its fertility rate (14). Incorporation of cholesterol to the sperm plasma membrane has been used to enhance the cryotolerance of sperm in many domesticated species (15–17). However, although sperm analytical parameters and fertility rates of donkey cooled semen were enhanced by the addition of CLC in that study (14), the overall pregnancy rates of mares inseminated with cooled jack semen remained low (47%) compared to the average pregnancy rates in clinical practice (60–70%) of mares inseminated with cooled horse stallion semen (18). Therefore, alternative methods are needed to augment the reproductive efficiency of donkeys.

The overall goal in this study was to develop approaches to optimize use of cooled donkey semen. The hypothesis was that donkey sperm extended in sodium casein-based or egg-yolk-based extenders have superior sperm analytical parameters and fertility rates compared to semen extended in skimmed milk-based extenders. This study aimed to (1) compare sperm traits (motility parameters, membrane integrity, mitochondrial membrane potential, production of reactive oxygen species) and fertility rates in mares inseminated with donkey semen extended in three different extender bases and cooled at 5°C, and (2) evaluate the effect of seminal plasma on these parameters.

## Materials and methods

This study was revised and approved by the Animal Care and Use Committee of São Paulo State University (UNESP), under protocol #0126/2019. Two experiments were carried out at the Department of Veterinary Surgery and Animal Reproduction, Faculty of Veterinary Medicine and Animal Science, UNESP Botucatu, SP, Brazil. Five out of the seven donkey jacks used in this study were owned by a private farm located in Laranjal Paulista, SP, Brazil. Owner consent was obtained prior to using the animals in this study.

### Experiment 1

#### Donkey semen cooling extended in skimmed milk, sodium caseinate, or egg-yolk based extenders

Seven Pêga donkey jacks (4–14 years old) were enrolled in this study. During the present study, all jacks were actively collected for breeding mares or jennies. Jacks were housed in stalls with free access to pasture. The animals were fed mixed alfalfa-grass hay and balanced grains, and had *ad libitum* water and mineral salt. Two washout semen collections were performed daily prior to the beginning of the study to standardize the extra-current ducts sperm reservoirs. Twenty-one (three per donkey) ejaculates were processed and used for the experiment. Semen collections were performed once a week on a jenny in estrus over three consecutive weeks. A rigid artificial vagina was used for semen collections. Immediately after collection, the semen was filtered to remove the gel fraction, and the gel-free volume was recorded. An aliquot (0.5 mL) of raw semen was diluted (1:1) in 10% formol saline and morphological characteristics were evaluated by differential interference contrast microscope (DIC, Leica DM 2500: Leica Microsystems, SP, Brazil) under 1,000 × magnification using oil immersion. Morphological characteristics were assessed in 100 sperm and abnormalities of the head, midpiece and tail were analyzed, resulting in the percentage of defects as previously described (19).

Sperm concentration was manually determined using the Neubauer chamber (Optik Labor, Lancing, England), under phase contrast microscopy (Jenamed 2 Zeiss: Carls Zeiss, Munich, Germany) at 200 × magnification, and semen was then split into six aliquots. Three out of the six samples were immediately diluted at 50 million sperm/mL in three different commercially available semen extenders: SM, skimmed milk-based extender containing CLC (BotuSemen Special^®^, Botupharma, Brazil); SC, sodium caseinate-based extender containing CLC (BotuSemen Gold^®^, Botupharma, Brazil); EY, egg yolk-based extender (BotuCrio^®^, Botupharma, Brazil). Extenders' compositions are highlighted in Table 1. The remaining aliquots (*n* = 3) were extended at 1:1 (v:v, semen:extender) with SM (*n* = 1) or SC (*n* = 2) and then processed using centrifugation (C, 600 × g/10 min). After centrifugation, the supernatant was discarded, and the sperm pellet of two out of the three samples was re-extended with the matched extender used pre-centrifugation (SM-C and SC-C). The extra sample previously diluted with SC was resuspended in EY after centrifugation (EY-C). After centrifugation, the sperm pellet was resuspended to 100 million sperm/mL. After extension, samples were stabilized for 20 min at room temperature (22°C), then stored in passive semen cooling containers (BotuFlex^®^, Botupharma, Brazil) at 5°C. Two units of the container were prepared for evaluations at 24 and 48 h post cooling, with one unit being opened at each timepoint. Sperm motility, plasma membrane integrity (PMI), plasma membrane stability (PMS), high mitochondrial membrane potential (HMMP), intracellular hydrogen peroxide (H_2_O_2_) and intracellular superoxide (${\text{O}}_{2}^{-}$) production were assessed at 0, 24, and 48 h after cooled storage.

**Table 1:** Composition of the extender used for cooling donkey semen.

**Extender**	**Composition**
BotuSemen Special^®^ (Botupharma, Brazil)	Skimmed milk-based extender (data provided: pH 6.8–7.2 and osmolarity 355–375) with undefined composition containing 20 g/L of skimmed milk, CLC, sugar, antioxidant, 1 g/L of gentamicin sulfate and 1 g/L of sodium penicillin.
BotuSemen Gold^®^ (Botupharma, Brazil)	Sodium caseinate-based extender (data provided: pH 6.8–7.2 and osmolarity 355–375) with undefined composition containing 20 g/L of sodium caseinate, CLC, sugar, antioxidant, 1 g/L of gentamicin sulfate and 1 g/L of sodium penicillin.
BotuCrio^®^ (Botupharma, Brazil)	Egg yolk-based extender (data provided: pH 6.8–7.2 and osmolarity 1,200) with undefined composition containing 10% of egg yolk, buffers, sugars, antioxidant, 500 mg/L of gentamicin, 1% of glycerol and 4% of formamid.

#### Sperm motility analyses

Sperm motility parameters were evaluated using computer-assisted sperm analysis (IVOS 12, Hamilton Thorne Inc., Beverly, MA, USA) using customized settings for equine sperm (20). For each sample, the percentiles of total motility (TM), progressive motility (PM), average path velocity (VAP, μm/s), straight-line velocity (VSL, μm/s), curvilinear velocity (VCL, μm/s), and rapid spermatozoa (RAP) were evaluated. Each sample was incubated in a dry bath at 37°C for 10 min before each evaluation. An aliquot was loaded in a Mackler chamber (Irvine Scientific, Santa Ana, CA), and a minimum of 1,000 cells in five random fields were assessed.

#### Flow cytometry analyses

The evaluation of the percentage of sperm with PMI, PMS, HMMP, intracellular H_2_O_2_ and ${\text{O}}_{2}^{-}$ production were carried out with LSR Fortessa equipment (Becton Dickinson, Mountain View, CA, USA) equipped with blue (488 nm, 100 mW), red (640 nm, 40 mW) and violet (405 nm, 100 mW) lasers (21, 22). For each assay, at least 10,000 cells per sample were analyzed and the data were extracted using the manufacturer's software (BD FACSDiva™ v6.1). For flow cytometry assays, all samples were extended in TALP-PVA containing Hoescht 33342 (7 μM in distilled water) to discard the non-cellular particles (23). The composition of the TALP-PVA medium was: 100 mM NaCl, 3.1 mM KCl, 25.0 mM NaHCO3, 0.3 mM NaH2PO4, 21.6 mM DL 60% sodium lactate, 2.0 mM CaCl2, 0.4 mM MgCl2, 10.0 mM Hepes-free acid, 1.0 mM sodium pyruvate, 1.0 mg/mL polyvinyl alcohol-PVA, and 25 μg/mL gentamicin, at a concentration of 5 × 10^6^ sperm/mL. Auto-fluorescence and controls of each fluorochrome were acquired for adjustment of wave overlap and compensation using the matrix compensation of the flow cytometer software.

Assessment of intracellular superoxide anion (${\text{O}}_{2}^{-}$) production, HMMP and PMS were carried out in association with 25 nM Yo-Pro^®^ (YP, Y3603 - Life Technologies; cell marking with plasma membrane instability), 20 μM MitoStatusRed (MSR, 564697- BD Pharmigen; mitochondrial potential) and 2 μM of Dihydroethidium (DHE, D23107 - Life Technologies; generation of intracytoplasmic superoxide anion). Briefly, 20 μM of MSR (diluted in DMSO), 25 nM of YP and 2 μM of DHE were added to 500 μL of semen solution containing 5 × 10^6^ sperm/mL, and incubated at 37°C for 20 min. For evaluation of intracellular H_2_O_2_, 2 μM of dihydrorhodamine 123 (D23806 - Life Technologies) was used in association with 1.5 μM of propidium iodide (diluted in TALP-PVA; cell marking with damaged plasma membrane). After reacting with ROS such as hydrogen peroxide or peroxide nitrite, dihydrorhodamine 123DHR is oxidized into a fluorescent compound (rhodamine 123), which is retained within the cell.

### Experiment 2

#### Fertility trial

Two Pêga donkey jacks (8 and 12 years old) belonging to the Department of Veterinary Surgery and Animal Reproduction, Faculty of Veterinary Medicine and Animal Science, UNESP Botucatu, SP, Brazil, enrolled in Experiment 1 had 131 ejaculates (Jack 1, 76 ejaculates; Jack 2, 55 ejaculates) collected for the fertility test. Both donkeys have been previously used for at least 4 years with known fresh semen fertility (~80% per cycle fertility rates). Semen collection and handling and sperm motility analysis were performed as previously described in Experiment 1.

The fertility test was carried out from September 2019 to April 2022. One hundred and sixty-three estrous cycles (Jack 1, *n* = 90; Jack 2, *n* = 73) were used to inseminate 15 crossbreed mares (10–11 cycles/mare), ranging from 5 to 15 years of age. The mares were kept on grass pasture, fed with silage, and supplemented with mineral salt. Mares were examined by transrectal ultrasonography (SonoScape A6^®^, Medical Corp, China) three times a week, and were administered prostaglandin F2alpha (dinoprost tromethamine, 5 mg/i.m., Lutalyse^®^, Zoetis, USA) to return to estrus when a corpus luteum was diagnosed. Once a preovulatory follicle (35 mm in diameter in the presence of endometrial edema) was detected, ovulation was induced with histrelin acetate (500 mg/i.m.; Strelin^®^, Butupharma, SP, Brazil). At 24 h post-induction of ovulation, all mares were artificially inseminated with 1 billion total sperm previously stored for 24 h at 5°C. All mares were randomly inseminated in a cross-over design with semen extended and cooled in one of the groups: SM, SC, EY, SM-C, SC-C, EY-C. Mares were examined 24 h after insemination to check for ovulation and the presence of free intrauterine fluid accumulation. If necessary, mares that had intrauterine fluid post-breeding were treated with oxytocin or uterine lavage. If a mare did not ovulate 24 h after breeding, the cycle was discarded, and the next cycle was used. Pregnancy diagnosis was performed at 15 days post-ovulation, and prostaglandin was administered to mares to return to estrus.

### Statistical analysis

Data analyses were carried out with GraphPad Prism 8.0.1.p. The Gaussian distribution was evaluated using the Shapiro-Wilk normality test. Semen parameters were assessed with a mixed model and Tukey's as a *post-hoc* test. Jacks were accounted as a random effect with ejaculate order, time of storage, and extender as fixed effects. A logistic regression model was used to evaluate pregnancy rates by considering pregnancy as the dependent variable and the treatment groups as explanatory variables. Significance was set at *P* ≤ 0.05 for all tests, and a statistically significant tendency was determined with 0.05 < *p* < 0.1. All data are presented as a mean ± SD.

## Results

### Experiment 1

**Figure 1:**
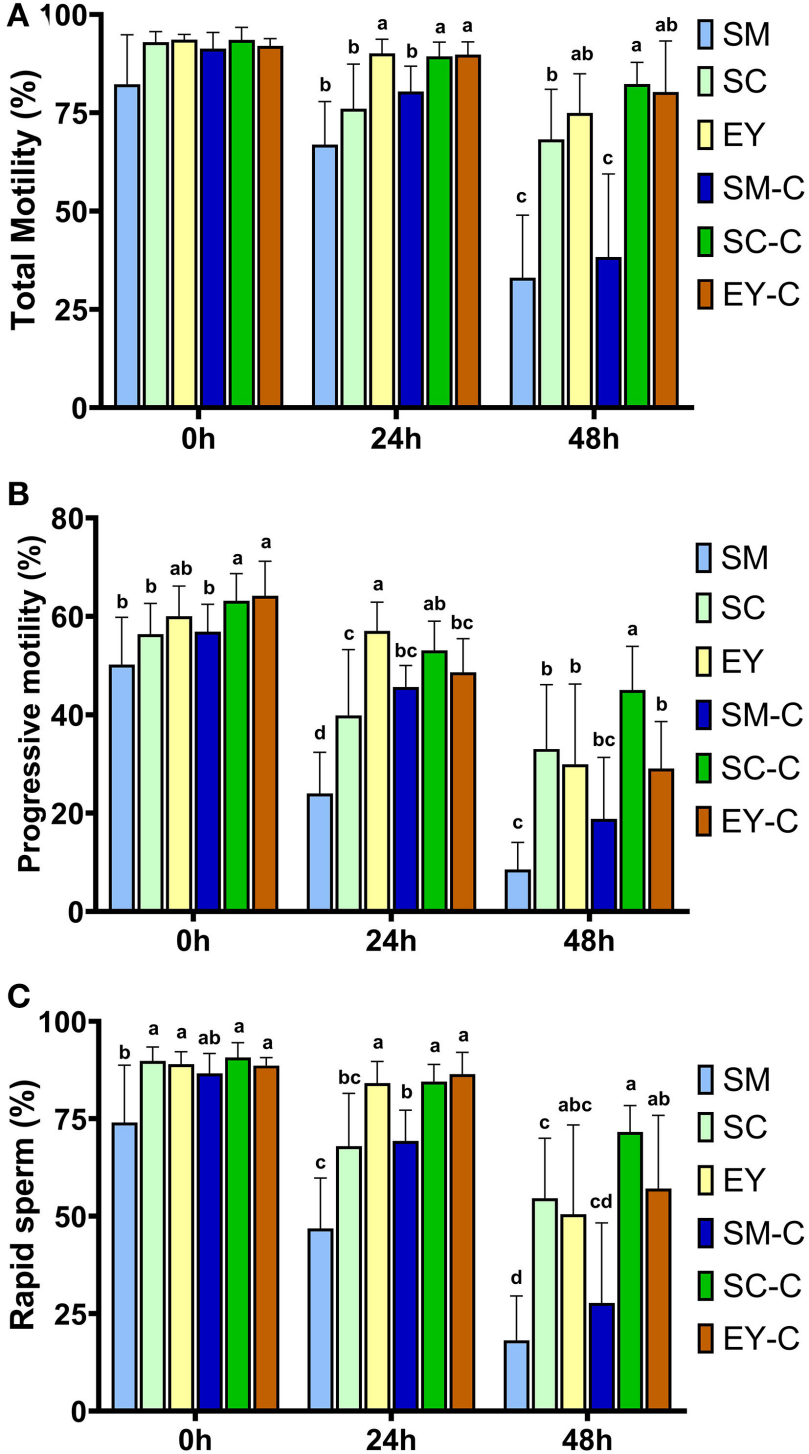
Sperm kinetics of donkey semen centrifuged **(C)** or not and extended in skimmed milk (SM), sodium caseinate (SC) or egg yolk (EY) based extender and cooled for 48 h. **(A)** Total sperm motility; **(B)** Progressive sperm motility; **(C)** Percentage of sperm with rapid movement. Different superscripts (^a, b, c, d^) denote differences between extenders (*P* < 0.05).

Ejaculates obtained from Pêga donkeys in Experiment 1 had the following characteristics (mean ± SD): gel-free volume 62.3 ± 29.9 mL, sperm concentration 344 ± 146 × 10^6^ sperm/mL, total sperm number 19.2 ± 7.7 × 10^9^, and morphological defects 10.2 ± 4.1%. Individual seminal characteristics of the jacks used for this Experiment are highlighted in Supplementary Table S1. There were no differences (*P* > 0.05) in TM between extenders at 0 h. However, the PM was superior in SC-C and EY-C to SM, SC, and SM-C (*P* < 0.05), while the EY group demonstrated intermediate results which did not differ from other groups (*P* > 0.05, Figure 1). In addition, the RAP was lower (*P* < 0.05) in SM than all SC and EY groups but not different (*P* > 0.05) to SM-C. The percentage of sperm with TM, PM, and RAP decreased over time in all groups (*P* < 0.05), except for TM in EY-C (*P* = 0.37). At 24 h, superior TM and RAP were achieved in groups EY, SC-C, and EY-C (*P* < 0.05). The PM of EY was superior to all other groups (*P* < 0.05), except for SC-C (*P* > 0.05). Semen diluted in SM had the lowest PM (*P* < 0.05), while semen in SM-C, SC, SC-C, and EY-C had intermediate values at this time (*P* > 0.05, Figure 1). After 48 h of cooling, both SM and SM-C groups had the lowest sperm kinetic parameters (P < 0.05). The SC-C produced superior PM to all groups (*P* < 0.05), as well as higher TM and RAP compared to SM, SC, and SM-C (*P* < 0.05), but not different to EY and EY-C (*P* > 0.05, Figure 1). The SC, EY, and EY-C showed intermediate PM values that did not differ from SM (*P* > 0.05, Figure 1).

Centrifugation did not affect (*P* > 0.05) sperm kinetics at 0 h in SM or EY and the TM and RAP in samples diluted with SC. However, a positive effect (*P* < 0.05) of centrifugation was observed for the percentage of sperm with PM at 0 h in semen centrifuged and re-extended in SC (Figure 1). In addition, semen centrifuged and re-extended in SC had higher (*P* < 0.05) TM, PM, and RAP at 24 and 48 h compared to non-centrifuged semen extended in SC (Figure 1). Centrifugation positively affected (*P* < 0.05) the PM and RAP at 24 h in semen extended in SM (Figure 1). Centrifugation did not affect (*P* > 0.05) TM and RAP in semen extended in EY; however, superior (*P* < 0.05) PM was observed in non-centrifuged semen extended in EY at 24 h. The results of VAP, VSL, and VCL are highlighted in Supplementary Table S2. Briefly, a reduction (*P* < 0.05) in all parameters occurred over time in all groups, and VAP, VSL, and VCL differed amongst groups (*P* < 0.05).

**Figure 2:**
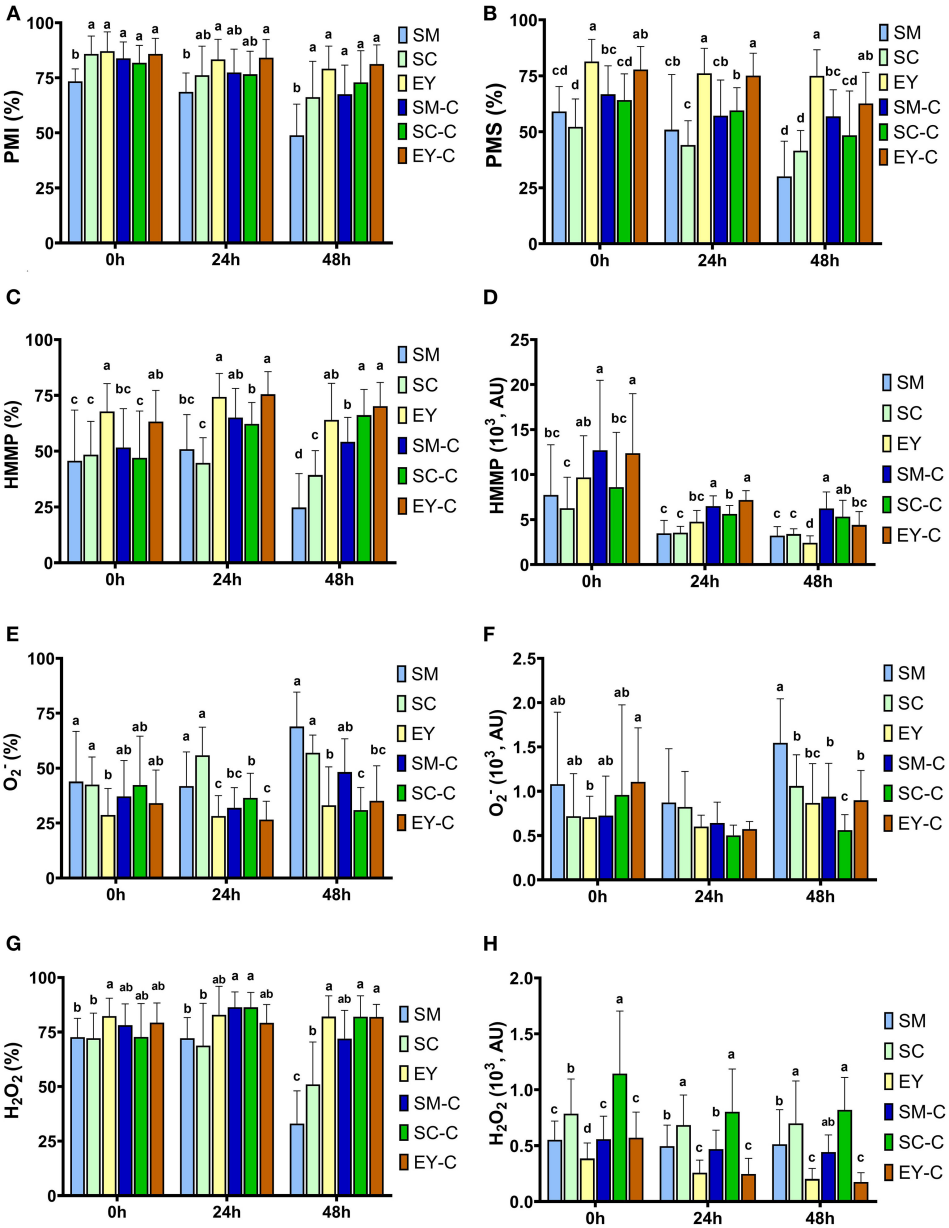
Flow cytometry analyses of donkey semen centrifuged **(C)** or not and extended in skimmed milk (SM), sodium caseinate (SC) or egg yolk (EY)-based extender and cooled for 48 h. **(A)** Percentage of sperm with intact plasma membrane (PMI); **(B)** Plasma membrane stability (PMS); **(C)** High membrane mitochondrial potential (HMMP); **(D)** Fluorescence intensity of light emitted from MitoStatusRed-stained sperm; **(E)** Sperm with high intracellular superoxide (${\text{O}}_{2}^{-}$); **(F)** Fluorescence intensity of light emitted from Dihydroethidium-stained sperm; **(G)** Sperm with high hydrogen peroxide (H_2_O_2_); **(H)** Fluorescence intensity of light emitted from Dihydrorhodamine-stained sperm. AU, arbitrary unit. Different superscripts (^a, b, c, d^) denote differences between extenders (*P* < 0.05).

Lower PMI was observed in SM group at 0 and 48 h (*P* < 0.05). At 24 h, SM had lower PMI than EY-C (*P* < 0.05), but similar PMI to all other groups (*P* > 0.05). No differences were found for PMI between the other groups (*P* > 0.05, Figure 2A). The PMI decreased (*P* < 0.05) over time in SM and SC groups, whereas semen in EY and EY-C groups had no differences in PMI over time (*P* > 0.05). Superior PMS was detected in semen samples extended in egg yolk-based extender (EY and EY-C) (*P* < 0.05), non-centrifuged semen extended in SM or SC exhibited the lowest values, and both SM-C and SC-C demonstrated intermediate values (Figure 2B). Similar to PMI, PMS decreased (*P* < 0.05) over time in SM and SC groups, whereas no difference was observed in cooled semen from EY and EY-C groups (*P* > 0.05).

The EY and EY-C groups exhibited superior HMMP to SM and SC groups at 0, 24, and 48 h (*P* < 0.05). Centrifuged semen extended in SM-based extender had similar HMMP up to 24 h than semen in EY-C and EY groups (*P* > 0.05). The semen from SC-C group exhibited lower HMMP up to 24 h when compared with both EY groups (*P* < 0.05), yet had similar results to both groups at 48 h (*P* > 0.05, Figures 2C,D). Intracellular O${}_{2}^{-}$ and H_2_O_2_ production were affected by the extender from 0 to 48 h, whereas centrifugation affected these parameters at 24 and 48 h in semen diluted in SM and SC (*P* < 0.05, Figures 2E–H). Semen extended in EY and EY-C had lower (*P* < 0.05) levels of O${}_{2}^{-}$ than semen from groups SM and SC, but did not differ from SM-C and SC-C (*P* > 0.05). Overall, H_2_O_2_ levels were higher in EY, EY-C, SM-C and SC-C than in semen from SM and SC.

### Experiment 2

Ejaculates used for the fertility trial had similar seminal parameters over time and between jacks (*P* > 0.05, Supplementary Table S3). Overall, as observed in Experiment 1, the sperm kinetic parameters of semen cooled for 24 h used for insemination were higher in samples extended in EY-based extender (*P* < 0.05), and the lowest sperm analytical parameters were observed in semen diluted in SM-based extender (*P* < 0.05, Table 2). Similarly, the SM group had lower fertility rates than SC, EY, SC-C, and EY-C (*P* < 0.05), while centrifuged semen extended in SM-based extender (SM-C) had similar fertility to SC (*P* > 0.05) that was lower than EY, SC-C, and EY-C (*P* < 0.05). The SC group had intermediate fertility which did not differ from the others (*P* > 0.05; Table 2). In addition, centrifugation tended to increase the fertility rate in semen extended in SM- and SC-based extenders (*P* = 0.09, Table 2). Overall fertility rates were similar between the two jacks (*P* > 0.05; Supplementary Table S4) and mares ovulated until 48 h after histrelin acetate treatment in 93% (163/175 cycles) of the cycles in the present study, and none presented anovulatory follicles.

**Table 2:** Sperm parameters and fertility rates of donkey semen centrifuged (C) or not and extended in skimmed milk (SM), sodium caseinate (SC) or egg yolk (EY)-based extender and cooled for 24 h.

**Group**	**TM**	**PM**	**RAP**	**Fertility**
SM	70.2 ± 6.7^c^	32.0 ± 4.7^d^	56.0 ± 9.5^c^	33% (9/27)^c^^*^
SC	80.0 ± 5.5^d^	40.4 ± 5.2^a^	67.2 ± 6.7^a^	67% (18/27)^ab^^*^
EY	87.2 ± 5.9^a^	41.0 ± 5.5^a^	73.9 ± 12.1^ab^	89% (25/28)^a^
SM-C	76.4 ± 6.2^bc^	35.2 ± 6.0^d^	61.1 ± 8.2^bc^	59% (16/27)^bc^^*^
SC-C	84.3 ± 4.5^ab^	48.2 ± 8.6^a^	74.8 ± 4.9^a^	89% (24/27)^a^^*^
EY-C	91.8 ± 3.0^a^	46.4 ± 6.1^a^	82.8 ± 7.5^a^	74% (20/27)^a^

TM, total motility; PM, progressive motility; RAP, percentage of rapid sperm. Different superscripts

(a, b, c, d) denote differences between groups (*P* < 0.05).

Asterisk

(*) denotes tendency (0.05 < *p* < 0.1) on the effect of centrifugation.

## Discussion

This study set forth to develop approaches to improve donkey semen cooling ability. To date, there is no gold standard protocol for cooling donkey semen, and clinical experience suggests that donkey semen has poor fertility and cooling ability when extended in commercially available equine SM- or milk-protein-based extenders (2). Even though some authors propose that this poor cooling ability may be circumvented by centrifuging jack semen to remove seminal plasma or adding 2% egg yolk to SM extenders, research is limited in this aspect and published results vary according to protocols (5, 7, 24–28).

Overall, the results of the present study showed that the sperm analytical parameters of cooled donkey semen were not preserved as well in SM-based extenders compared to EY-based and SC-based extenders containing CLC. The preservation of superior sperm analytical parameters in EY- and SC- groups were not surprising to the authors as egg yolk is known to be beneficial to donkey sperm analytical parameters (2, 5, 7, 27), and INRA 96, a commercially available semen extender based on a defined milk protein (e.g., native phosphocaseinate), has been reported to maintain the motility of cooled donkey semen for longer periods than a SM-based extender (8). In contrast, the comparable sperm analytical parameters between semen extended and cooled in SC and semen cooled in EY were unexpected because a prior study showed that the inclusion of 2% egg yolk improved the cooling ability of donkey semen extended in INRA 96 (7). It is possible that the similar results yielded by both SC and EY groups in the present study may be associated with inclusion of CLC or sodium caseinate (a different milk protein from that present in INRA 96) in the extenders used in this study. Arguably, the native phosphocaseinate present in INRA 96 is a similar molecule to sodium caseinate and may have reasonably provided equivalent sperm protection during cooling (29). Therefore, the authors postulate that the addition of CLC in the SC-based extender used herein was more likely to have contributed to the comparable results between SC and EY groups. Furthermore, inclusion of cholesterol to extenders has been reported to be beneficial to cooled donkey sperm (14). Further, although the cooling tolerance of donkey semen in SC extenders has not yet been evaluated, CLC and SC was found to have improved sperm characteristics for horse stallion semen when compared with INRA 96 in one study (29).

Inclusion of CLC in semen extenders has been used over the years to increase sperm membrane resistance to cryopreservation processes in many species, including donkeys (15–17, 30, 31). Sperm cryotolerance is reliant on the plasma membrane cholesterol:phospholipid ratio, which maintains fluidity and stability of the sperm membrane at low temperatures (15–17, 30–32). Specifically in donkeys, CLC improved sperm cryo-resistance and post-thaw sperm analytical parameters (30, 31), and cooling ability (14). Interestingly, there was no improvement in the fertility of frozen-thawed donkey semen in horse mares in one study (30), but the inclusion of CLC in milk-based extender improved fertility rates in mares inseminated with cooled donkey semen (14). It is worth noting that the amount of CLC added in the study evaluating the fertility of post-thawed donkey semen in horse mares (30) exceeded the minimal threshold of plasma membrane cholesterol, which presumably inhibited sperm capacitation. The efflux of cholesterol from the plasma membrane is critical for sperm capacitation and fertilization (33), and excessive cholesterol levels in the sperm plasma membrane are well known to impair sperm capacitation and acrosome reaction (32, 34, 35). However, it is worth noting that the SM-based extender containing CLC used in the present study was previously tested in jacks (14), and both SM- and SC-based extenders containing CLC used in the present study were tested in horse stallions with no impairment to fertility (29, 36, 37). Therefore, it was reasonable to speculate that the extender containing CLC used in the present study would not impair the sperm capacitation process and fertility of cooled donkey semen.

Clinical experience and prior studies have suggested that donkey sperm needs an additional source of cholesterol (2, 4, 5, 7, 11), and the addition of egg yolk to SM-based extenders as a cholesterol source has been previously used to mitigate the poor cooling ability of donkey semen (2). Therefore, the superior sperm analytical parameters observed in donkey semen extended in EY-based extender in this study did not come as a surprise to the authors. Donkey semen has demonstrated to be better preserved at 5°C for 72 h in INRA 82 with 2% egg yolk than in INRA 82 and INRA 96 (7). Similarly, Baken's extenders containing 3 or 10% of egg yolk was reported to better preserve sperm analytical parameters of donkey semen after cooling at 5°C compared with Kenney extender (5, 27), and the higher concentration of egg yolk (10%) was preferred for donkey sperm cooled in Baken's extender (4). In addition, commercially available semen extenders with non-declared proprietary compositions containing buffers, milk, and egg yolk (e.g., Gent extender) (28), or purified proteins and a mixture of low-molecular-weight lipids (e.g., Hippex) also seem to better preserve of donkey sperm analytical parameters for up to 72 h at 4–5°C compared to milk- (e.g., Kenney) or purified milk protein-based (e.g., INRA 96 and Equiplus) extenders (11, 38). Remarkably, the EY extender used in the present study for cooling donkey semen is a commercially available freezing extender, which may come as a surprise for some practitioners and researchers as the cryoprotectants (e.g., glycerol, formamide) contained in semen freezing extenders have been proposed to be toxic to stallion and jack sperm (10, 24, 39). However, the EY extender yielded better sperm analytical parameters and fertility in cooled donkey semen than the SM extender in the present study. The authors assume that these results might be associated with the lower concentration of glycerol in the extender used in this study (1% of glycerol and 4% of formamide), which is an ~5-fold less than the reported spermatotoxic concentration for horse and donkey sperm (5% of glycerol) (10, 24).

Centrifugation and removal of seminal plasma is another prevalent recommendation for cooling donkey semen (2). In the present study, seminal plasma removal improved sperm analytical parameters and fertility of donkey semen extended in SM- and SC-based extenders, but not in EY-based extenders. The reason for decreased cooling tolerance of donkey semen containing seminal plasma in milk-based extenders is not well understood. Although recent studies on donkey seminal plasma compounds and effects have suggested an important interaction between the seminal plasma composition in donkeys and the sperm freezing ability (40, 41), these interactions have not yet been described with regards to how well donkey sperm tolerates cooling. It has been suggested that donkey seminal plasma contains proteins that remove cholesterol from the sperm plasma membrane (2). In effect, the seminal plasma composition in donkeys differs from horses (42, 43), as donkey seminal plasma contains 4–10 times higher protein content, and other metabolites such as glucose, calcium, lipids, and phosphorus compared to horse stallions' seminal plasma (42). However, although centrifugation improved the ability of donkey semen extended in SM to tolerate cooling in the present study, it did not yield comparable sperm analytical parameters and fertility to SC- and EY-extended semen. Interestingly, Carvalho et al. reported that the sperm-rich fraction of jack semen cooled in a SM-based extender resulted in superior motility parameters and fertility in horse mares compared to a lactose-egg yolk extender (13). These results may illustrate the interaction between the seminal plasma and sperm cooling tolerance with SM extenders in donkeys. However, although centrifugation improved the sperm parameters and fertility of semen extended in SM and SC, those were not superior to EY groups in the present study. Of note is that semen was not extended in an EY-based extender pre-centrifugation in EY-C groups but in SC-based extender. This approach was elected by the authors to reflect practical cost-considerations in the field as semen freezing extenders (i.e., EY-based) are more expensive than cooling extenders. In addition, it is worth noting that all centrifuged semen samples were resuspended at higher sperm concentrations (100 million sperm per mL) than semen not submitted to centrifugation (50 million sperm per mL). This approach has been suggested by the manufacturer of the extender to avoid exceeding the minimal threshold of plasma membrane cholesterol (32, 34, 35) and was described in one study evaluating SC-based extender containing CLC (29).

The findings in this study also corroborate with the strong correlation previously reported between PMI, HMMP, and sperm motility (44). EY-based extender was observed to better preserve donkey sperm PMI, PMS, HMMP, and motility characteristics; and removal of seminal plasma positively affected these parameters in semen extended in SM and SC. In addition, there were lower levels of ${\text{O}}_{2}^{-}$ and higher levels of H_2_O_2_ in semen from EY groups, SM-C, and SC-C. Prior studies have indicated an association between HMMP levels and ${\text{O}}_{2}^{-}$ production (45, 46). Although ${\text{O}}_{2}^{-}$ is the major and first byproduct of sperm metabolism, this molecule can also be generated from membrane-associated NADPH oxidase (47). Superoxide anion is the precursor for most reactive oxygen species (ROS) and is poorly lipophilic, acting only at the site of production (48). However, ${\text{O}}_{2}^{-}$ radicals can be involved in the Haber-Weiss' reaction with H_2_O_2_ to form hydroxyl radicals. The hydroxyl radical is considered one of the most potent oxidizing agents, and can cross membranes to react with molecules in the unsaturated lipids in cell membranes and DNA (48, 49). Sperm cryoinjuries are associated with overproduction of ROS (50–52) and subsequent lipid peroxidation (LPO) of the plasma membrane (51, 53, 54). In effect, donkey sperm is especially sensitive to these events due to the large amount of polyunsaturated fatty acids in their plasma membrane (53, 55). Lipid peroxidation is triggered by OH reactions, in which H^+^ is sequestered from the polyunsaturated fatty acids present in the phospholipid bilayer of the sperm membranes and subsequently giving rise to alkoxyl (RO•) or peroxyl (ROO•) radicals, which tend to stabilize by withdrawing H^+^ and an electron present in the adjacent phospholipids, generating a chain reaction and culminating in damage of the plasma membrane (56). Although LPO was not assessed in the present study, the higher levels of ${\text{O}}_{2}^{-}$ observed in semen samples extended and cooled in SM- and SC-based extenders might be associated with the higher PMI and PMS changes observed in these groups, which might be associated with higher LPO in these groups. In contrast, H_2_O_2_ is not considered a free radical because of its chemical stability (48). Hydrogen peroxide is present in high concentrations in the seminal plasma of stallions with good semen quality and has been proposed to have a positive correlation with normal sperm metabolism (57). It has also been suggested that H_2_O_2_ production is simply a byproduct of high mitochondrial activity, with ROS levels positively correlated to fertility rate in stallions (57), as was observed in the present study. Of interest, a recent study has reported differences in the antioxidant composition and effect of donkey seminal plasma when compared to horses (43). Donkeys present higher levels of enzymatic antioxidants (e.g., superoxide dismutase, SOD; catalase, CAT; glutathione peroxidase, GPX; and glutathione reductase, GSR) than horses, and a positive interaction of CAT and SOD with sperm motility has been observed in donkeys but not in horses (43). In addition, higher levels of enzymatic antioxidants (e.g., SOD, CAT, GPX, GSR, and paraoxonase type 1) and non-enzymatic antioxidant components (measured in terms of copper-reducing antioxidant capacity, ferric-reducing ability of plasma, and Trolox equivalent antioxidant capacity) have also been associated with sperm cryotolerance in donkeys (43, 55, 58). However, although physiologically the seminal plasma represents the most important source of enzymatic and non-enzymatic antioxidants for sperm, being capable of eliminating the excess of ROS that induces oxidative stress to sperm (43, 53, 55, 59), the seminal plasma has harmful effects on equids' sperm and must be removed prior to freezing (50, 60). Some authors assume that the brief contact of sperm with seminal plasma or supplemented antioxidants before their removal may be sufficient to exert the beneficial effect of antioxidants on sperm cryotolerance (20, 53, 55). In addition, it is worth mentioning that most commercially available semen extender, including the ones used in the present study, have antioxidants in their composition. Supplementation of semen with antioxidants allied with removal of the seminal plasma has been associated with the increment on sperm viability after cooling or freezing (60). Although it is widely recognized that high levels of ROS can cause sperm damage, moderate concentrations of ROS play an essential role for destabilization of the sperm membrane, sperm capacitation, and fertility potential (48, 61). Therefore, an adequate balance between ROS and antioxidant levels is essential for optimal sperm function (62).

Even though some studies have reported fertility rates of cooled donkey semen ranging from 21 to 76% using extenders with different bases, the average period of storage in the majority of studies has been short (e.g., 2–8 h) (24–26) or mares were inseminated several times in the same estrous cycle (13, 63). Therefore, in our previous study with cooled donkey semen (14), the authors deliberately mimicked conditions in equine practice where semen is often shipped over long distances for 24–48 h (2). To the best of our knowledge, that was the first study assessing the fertility of mares inseminated once in the estrus cycle with donkey semen cooled-stored for a longer period (24 h). It is worth noting that even though our previous study showed that inclusion of CLC in a SM-based extender could improve the fertility of cooled donkey semen in horse mares (14), the fertility rates of mares bred with cooled donkey semen (47%) were below the average of mares inseminated with cooled horse stallion semen (60–70%) (18). Fortunately, the present study demonstrated two methods to circumvent this poor ability of donkey semen to tolerate cooling (extending the semen in EY-based extender, or removing the seminal plasma and extending in SC-based extender) which achieved satisfactory pregnancy rates (74–89%) in mares (18). However, it is also essential to recognize that even though centrifugation and removal of seminal plasma have been a prevalent recommendation for cooling donkey semen (2), seminal plasma appears to play an essential role in the modulation of the endometrial response after artificial insemination in jennies (40, 64, 65). Seminal plasma decreases sperm binding to polymorphonuclear (PMNs) and other inflammatory cells to sperm (66, 67), and more specifically in donkey species, seminal plasma has been described to suppress the *in vitro* sperm-attachment of PMNs collected from uterine secretions of jennies after artificial insemination (40, 64), downregulate endometrial COX2 expression in jennies upon artificial insemination with frozen-thawed semen (68), induce neutrophil extracellular traps (NETs) formation (69), and increase pregnancy rates following artificial insemination with frozen-thawed semen in this species (70); even though the addition of seminal plasma to frozen-thawed donkey semen samples does not improve *in vitro* sperm characteristics (71). Therefore, even though fertility rates were improved in mares by removal of seminal plasma in semen sample diluted in SM- or SC-based extenders in the present study, fertility rates may be impaired in jennies by the lack of seminal plasma.

In conclusion, CLC-containing SC-based and EY-based extenders seemed to better preserve the sperm characteristics and fertility of cooled donkey semen. In addition, removal of seminal plasma *via* centrifugation is a viable alternative to augment sperm analytical parameters of donkey semen cooled and extended in SM or milk protein-based extenders. The *in vitro* benefits of SC and EY on semen parameters were translated into *ex vivo* improved fertility of cooled donkey semen in horse mares. However, more studies are needed to determine if similar results can be obtained in jennies.

## Data availability statement

The raw data supporting the conclusions of this article will be made available by the authors, without undue reservation.

## Ethics statement

The animal study was reviewed and approved by Animal Care and Use Committee of São Paulo State University. Written informed consent was obtained from the owners for the participation of their animals in this study.

## Author contributions

MG, VS, and RB contributed to study execution. LS contributed to study design and execution, data analysis, interpretation, and preparation of the manuscript. CF-D contributed to study execution and preparation of the manuscript. JD and FP contributed to the study design and preparation of the manuscript. All authors gave their final approval of the manuscript.

## Funding

The authors would like to thank the São Paulo Research Foundation (FAPESP) grant #2017/13883-9, and CAPES (Coordination for the Improvement of Higher Education Personnel) for financial support.

## Conflict of interest

The authors declare that the research was conducted in the absence of any commercial or financial relationships that could be construed as a potential conflict of interest.

## Publisher's note

All claims expressed in this article are solely those of the authors and do not necessarily represent those of their affiliated organizations, or those of the publisher, the editors and the reviewers. Any product that may be evaluated in this article, or claim that may be made by its manufacturer, is not guaranteed or endorsed by the publisher.
