# Supplementary material for: Ability of donkey sperm to tolerate cooling: Effect of extender base and removal of seminal plasma on sperm parameters and fertility rates in mares
Source: Front Vet Sci. 2022 Sep 26;9:1011899. doi: 10.3389/fvets.2022.1011899 (PMC9548546; doi:10.3389/fvets.2022.1011899)
Supplement: Supplementary file 1 [file Data_Sheet_1.docx]

Supplementary Material

**Table S1.** Seminal characteristics of three ejaculates of seven Pêga jacks.

| Ejaculate order | Gel free volume  (mL) | | Concentration  (×10^6^/mL) | | Total sperm ejaculated  (×10^9^/mL) | | Morphological defects (%) | |  |
| --- | --- | --- | --- | --- | --- | --- | --- | --- | --- |
|  | Mean ±SD | Range | Mean ±SD | Range | Mean ±SD | Range | Mean ±SD | Range |  |
| **1** | 54.3 ± 9.1 | 46 – 64 | 271.7 ± 132.5 | 165 – 420 | 15.6 ± 10.1 | 7.6 – 26.9 | 11.3 ± 3.2 | 9 – 15 |  |
| **2** | 77.3 ± 18.0 | 60 – 96 | 196.7 ± 18.9 | 175 – 210 | 15.4 ± 4.6 | 10.5 – 19.7 | 12.7 ± 2.1 | 11 – 15 |  |
| **3** | 74.0 ± 66.1 | 30 – 150 | 449.0 ± 259.2 | 150 – 610 | 21.9 ± 4.0 | 17.6 – 25.6 | 11.7 ± 6.7 | 6 – 19 |  |
| **4** | 40.3 ± 15.6 | 24 – 55 | 379.0 ± 25.9 | 350 – 400 | 15.5 ± 6.5 | 8.4 – 21.3 | 10.0 ± 2.0 | 8 – 12 |  |
| **5** | 56.0 ± 20.9 | 32 - 70 | 466.7 ± 51.1 | 430 - 525 | 26.6 ± 11.7 | 13.7 – 36.8 | 6.7 ± 2.1 | 5 – 9 |  |
| **6** | 84.0 ± 29.5 | 52 – 110 | 204.7 ± 17.5 | 187 – 222 | 17.4 ±6.8 | 9.7 – 22.6 | 10.3 ± 7.6 | 5 – 19 |  |
| **7** | 50.3 ± 16.5 | 32 – 64 | 443.3 ± 32.2 | 420 – 480 | 22.3 ± 7.4 | 13.8 – 26.9 | 8.7 ± 2.3 | 6 – 10 |  |

Supplementary Material

**Table S2.** Sperm parameters of donkey semen centrifuged or not and extended in skimmed milk (SM), sodium caseinate (SC) or egg yolk (EY)-based extender and cooled for 48 h.

|  |  | **Non-centrifuged** | | |  | **Centrifuged** | | |
| --- | --- | --- | --- | --- | --- | --- | --- | --- |
|  |  | **SM** | **SC** | **EY** |  | **SM** | **SC** | **EY** |
| **0 h** | **VAP** | 125.8±3.2^Xc^ | 143.8±1.9^Xa^ | 119.2±2^Xd^ |  | 139.9±2.2^Xb^ | 139.9±1.2^Xb^ | 123.3±1.9^Xcd^ |
|  | **VSL** | 102.9±2.9^Xcd^ | 115.6±1^Xab^ | 97.4±1.8^Xd^ |  | 113.5±1.7^Xb^ | 117.2±1.4^Xa^ | 103.6±1.1^Xc^ |
|  | **VCL** | 225.8±4.5^Xb^ | 240.8±4.3^Xa^ | 204.4±3.3^Xd^ |  | 240.7±3.6^Xa^ | 238±2.4^Xa^ | 211.3±3.7^Xc^ |
|  |  |  |  |  |  |  |  |  |
| **24 h** | **VAP** | 88.2±2.7^Yd^ | 119.4±3.4^Yb^ | 111.3±2.6^Yc^ |  | 117.6±2.6^Yb^ | 128.8±1.3^Ya^ | 117.2±1.4^Yb^ |
|  | **VSL** | 66.9±2^Yd^ | 94±3.5^Yb^ | 91.1±1.8^Yc^ |  | 95.3±2^Yb^ | 103.1±1.6^Ya^ | 90.7±1.1^Yc^ |
|  | **VCL** | 173±4.3^Ye^ | 217.4±5.2^Yb^ | 197±4.3^Yd^ |  | 211.9±4.2^Yc^ | 228.9±2.2^Ya^ | 210.2±2.2^Xc^ |
|  |  |  |  |  |  |  |  |  |
| **48 h** | **VAP** | 78.5±3.1^Ze^ | 117.1±3.5^Za^ | 83.7±4.6^Zd^ |  | 92.5±6.3^Zc^ | 111.5±3.1^Zb^ | 85.2±3.4^Zd^ |
|  | **VSL** | 58.9±2.4^Zd^ | 91±2.4^Za^ | 65.2±4^Zc^ |  | 76.2±5^Zb^ | 89±3.3^Za^ | 65.2±2.3^Zc^ |
|  | **VCL** | 158.4±5.5^Zd^ | 214.2±6^Za^ | 164.2±7^Zc^ |  | 174.1±10.7^Zc^ | 203.8±5.1^Zb^ | 169.1±5.5^Yc^ |

VAP, average path velocity (µm/s); VCL, curvilinear velocity (µm/s); VSL, straight-line velocity (µm/s); Different superscripts denote differences within (^X,Y,Z^) columns for the same variable, and different lowercase letters (^a,b,c,d^) denote difference between columns (*P*<0.05).

Supplementary Material

**Table S3.** Seminal parameters of two Pêga jacks used for the fertility trial (mean±SD).

| **Jack** | Gel free volume  (mL) | | Concentration  (×10^6^/mL) | | Total sperm ejaculated  (×10^9^/mL) | | Total motility (%) | | Progressive motility (%) | | Rapid sperm (%) | |
| --- | --- | --- | --- | --- | --- | --- | --- | --- | --- | --- | --- | --- |
|  | Mean±SD | Range | Mean±SD | Range | Mean ±SD | Range | Mean ± SD | Range | Mean ± SD | Range | Mean ± SD | Range |
| **1** | 27.0 ± 11.5 | 10 – 50 | 417.5 ± 115.9 | 210 – 680 | 10.4 ± 3.5 | 4.8 – 16.3 | 92.9 ± 2.7 | 88 – 96 | 64.2±6.0 | 50 – 72 | 87.3±4.3 | 79 – 94 |
| **2** | 51.1 ± 24.4 | 25 – 100 | 568.5 ± 313.8 | 170 – 1200 | 21.2 ± 8.4 | 7.0 – 34.2 | 91.6 ± 4.5 | 83 – 97 | 61.4 ± 11.8 | 30 – 75 | 88.5 ± 4.6 | 80 – 95 |
| **Overall** | 34.7 ± 19.9 |  | 465.5 ± 209.5 |  | 13.8 ± 7.5 |  | 92.5 ± 3.4 |  | 63.3 ± 8.2 |  | 87.6 ± 4.3 |  |

**Table S4.** Fertility rates of donkey semen centrifuged (C) or not, extended in skimmed milk (SM), sodium caseinate (SC) or egg yolk (EY)-based extender, and cooled for 24 hours.

| **Jack** | **SM** | **SC** | **EY** | **SM-C** | **SC-C** | **EY-C** | **Overall** |
| --- | --- | --- | --- | --- | --- | --- | --- |
| **1** | 27% (4/15)^b^ | 73% (11/15)^a^ | 93% (14/15)^a^ | 60% (9/15)^ab^ | 93% (14/15)^ab^ | 73% (11/15)^ab^ | **70% (63/90)** |
| **2** | 41% (5/12)^b^ | 58% (7/12)^ab^ | 84% (11/13)^a^ | 58% (7/12)^ab^ | 83% (10/12)^ab^ | 75% (9/12)^ab^ | **67% (31/73)** |

Different superscripts (^a,b,c,d^) denote differences between groups (*P*<0.05).
